# Multigenerational genetic inheritance and clinical characteristics of the rare disease hypophosphatasia in 6 families: A case series

**DOI:** 10.1016/j.bonr.2025.101857

**Published:** 2025-07-15

**Authors:** Peter Kannu, Aliya A. Khan, Mira Francis, Jonathan D. Adachi

**Affiliations:** a University of Alberta, 8613 114 Street, Edmonton, AB T6G 1C9, Canada; b McMaster University, 1280 Main St W, Hamilton, ON L8S 4L8, Canada; c Alexion, AstraZeneca Rare Disease, 1004 Middlegate Road, Mississauga, ON L4Y 1M4, Canada

**Keywords:** Hypophosphatasia genotype, Phenotype, Alkaline phosphatase, Family mapping

## Abstract

Family mapping is a useful tool for tracking the inheritance of rare inherited diseases, including hypophosphatasia (HPP), through generations. We show the inheritance of HPP in 6 affected families, describing genetic variants, biochemical hallmarks, and clinical manifestations among family members. Mapping families with HPP is warranted in clinical practice to better understand monitoring needs for potentially affected individuals over time, since manifestations of HPP can arise throughout a patient's lifespan.

## 1 Introduction

Hypophosphatasia (HPP) is a rare, inherited disease typically caused by genetic variants in tissue-nonspecific alkaline phosphatase (ALP, gene name *ALPL*) that lead to deficient enzyme activity (Del Angel et al., 2020; Salles, 2020). Deficient tissue-nonspecific ALP activity can cause extracellular accumulation of its substrates, including vitamin B_6_, which may be used to support a diagnosis of HPP (Salles, 2020; Khan et al., 2024). The estimated prevalence of HPP for patients with early-onset disease that first manifests in infants <6 months of age is 1 in 300,000 births (Mornet et al., 2011; Whyte et al., 2015).

HPP is characterized by an array of skeletal and nonskeletal manifestations that can vary widely between patients, including patients within the same family (Kishnani et al., 2017; Huggins et al., 2020). In children, common features of HPP include rickets, fractures, premature tooth loss, waddling gait, delayed walking, muscle weakness, and nephrocalcinosis; in adults, hallmark manifestations include poorly healing or recurrent fractures, chronic musculoskeletal pain, abnormal dentition (including tooth loss), muscle weakness, and fatigue (Kishnani et al., 2017). Routine clinical assessment is particularly important in patients with HPP, because manifestations may evolve and accumulate throughout their lifespan (Szabo et al., 2019).

The high level of clinical heterogeneity in HPP is typically attributed to the >460 *ALPL* variants that have been identified to date, although the overall correlation between genotype and phenotype in HPP is limited (Del Angel et al., 2020; Farman et al., 2024; Mornet, 2018). This retrospective observational study used patient records from 6 families with a history of HPP who were seen at 3 sites in Canada. *ALPL* variants and clinical characteristics of multigenerational family members were summarized descriptively. The objective of the study was to assess genetic inheritance and clinical characteristics of HPP in affected families. In this case series, the term “asymptomatic carrier” is used to describe individuals who had an *ALPL* variant but no biochemical or clinical signs of disease. The term “subclinical HPP” is used to describe individuals who had an *ALPL* variant and the biochemical signature of HPP (low ALP activity and/or elevated vitamin B_6_ concentration) without overt clinical manifestations of the disease. The results of this case series demonstrated the heterogeneity of clinical HPP manifestations between and within families and highlight the need to perform family mapping to assess potentially affected individuals over time.

## 2 Cases

### 2.1 Family 1

**Fig. 1:**
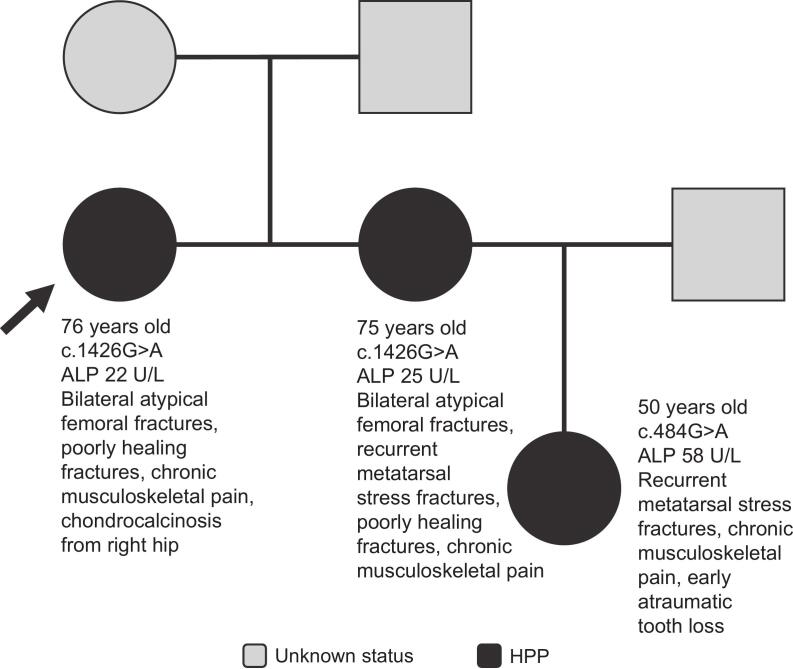
Family 1 pedigree. Patient age at time of assessment, *ALPL* variant, ALP activity, and clinical manifestations are shown on the pedigree for each patient. Arrow indicates proband.

The proband is a 76-year-old woman who was 154 cm tall and weighed 71 kg at the time of assessment (Fig. 1). She presented with chronic musculoskeletal pain, chondrocalcinosis in the right hip, and osteoporosis (femoral neck bone mineral density T-score: −2.6). She had a history of bilateral atypical femoral fractures and poorly healing fractures. Fractures of the pelvis, wrist, and ankle were recorded. Her ALP activity was 22 U/L (low ALP in adults defined as <40 U/L). Genetic testing revealed a heterozygous c.1426G>A variant in *ALPL*. This proband was diagnosed with HPP based on clinical, biochemical, and genetic findings.

The same heterozygous *ALPL* variant was detected in the proband's sister, a 75-year-old female (height, 150 cm; weight, 52 kg). Her ALP activity was 25 U/L, and her vitamin B_6_ level was 193 nmol/L (reference range: 20–96 nmol/L). She had a history of clinical manifestations similar to those of the proband, including bilateral atypical femoral fractures, recurrent metatarsal stress fractures, chronic musculoskeletal pain, and osteoporosis (femoral neck bone mineral density T-score: −3.4). Her fractures were poorly healing. Similar to the proband, she was diagnosed with HPP based on clinical, biochemical, and genetic findings.

The proband's niece also had the same heterozygous *ALPL* variant (c.1426G>A). This niece had a history of major dental problems, early atraumatic tooth loss, recurrent metatarsal stress fractures, and chronic musculoskeletal pain. Additional fractures in her fingers and nose were reported. Her ALP activity was not low (58 U/L), but she was diagnosed with HPP based on clinical manifestations and genetic test results. Notably, this patient had a history of fatty liver disease, which may have increased her serum ALP activity (Van Velsen et al., 2023).

### 2.2 Family 2

**Fig. 2:**
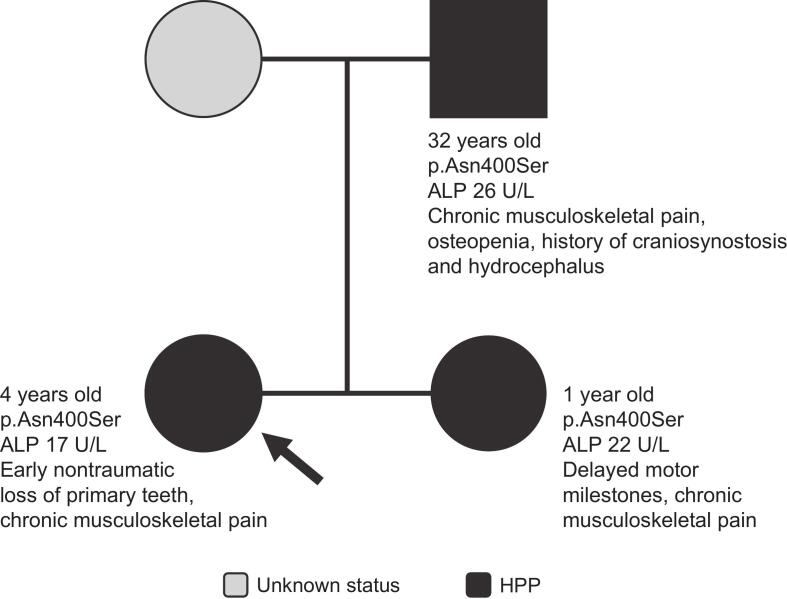
Family 2 pedigree. Patient age at time of assessment, ALP variant, and clinical manifestations are shown on the pedigree for each patient. Arrow indicates proband.

The proband is a 4-year-old girl who experienced early nontraumatic loss of her primary teeth at age 2 years (Fig. 2). Her vitamin B_6_ level was 45.6 μg/L (reference range: 2.0–32.8 μg/L), and her ALP activity was low (17 U/L). The proband was found to have a heterozygous *ALPL* variant resulting in a p.Asn400Ser protein mutation and was diagnosed with HPP at 4 years of age. The proband has a documented medical history spanning 24 years of follow-up. Now 28 years old, she reported chronic joint and bone pain starting at age 11 years and was diagnosed with depression and anxiety at age 20 years.

After the proband's diagnosis, family screening was performed. Her father (now 55 years old; aged 32 years at time of HPP diagnosis) had a history of craniosynostosis as an infant, hydrocephalus as a child (requiring a shunt), osteopenia, chronic musculoskeletal pain, and muscle spasms in his legs, midback, and hands. His ALP activity was low (26 U/L), and he was found to have the same *ALPL* variant as his daughter. He was diagnosed with HPP based on genetic and biochemical findings, as well as medical history. The proband's sister was diagnosed with HPP at age 1 year after genetic testing revealed the same heterozygous variant as the proband. Her vitamin B_6_ level was 69.0 μg/L (reference range: 2.0–32.8 μg/L), and her ALP activity was 22 U/L. She had a history of recurrent vomiting throughout childhood and had a learning disability that affected her reading and writing. From age 17 to 20, she experienced chronic musculoskeletal pain and generalized fatigue, and at age 20, she began experiencing headaches occurring once or twice a week. No data are available for the proband's mother.

### 2.3 Family 3

**Fig. 3:**
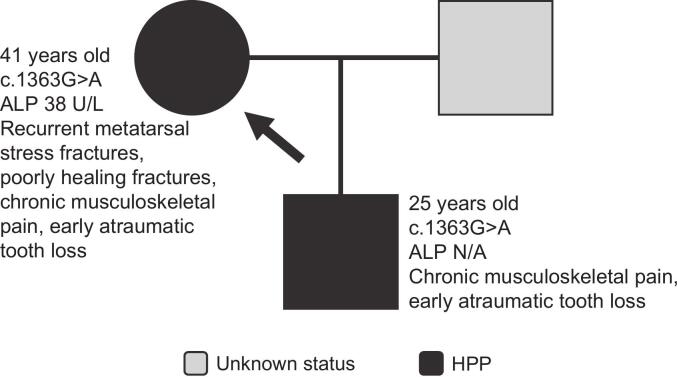
Family 3 pedigree. Patient age at time of assessment, *ALPL* variant, ALP activity (if known), and clinical manifestations are shown on the pedigree for each patient. Arrow indicates proband.

The proband is a 41-year-old woman who was 159 cm tall and weighed 44 kg at the time of assessment (Fig. 3). She had a history of recurrent metatarsal stress fractures, chronic musculoskeletal pain, osteoporosis (femoral neck bone mineral density T-score: −3.0), and tailbone fracture. Her fractures were poorly healing. She also had a history of early atraumatic tooth loss. Her ALP activity was 38 U/L. Genetic testing revealed the presence of a heterozygous c.1363G>A variant in *ALPL*, and she was diagnosed with HPP based on collective findings. The proband's son, a 25-year-old, was found to have the same heterozygous variant. He was 165 cm tall and weighed 48 kg at time of assessment. He had a history of seizures, early atraumatic tooth loss, chronic musculoskeletal pain (growing pains, polyarthralgia) and osteopenia (femoral neck bone mineral density T-score: −2.2). Although his ALP activity was not recorded, he was diagnosed with HPP based on genetic and clinical findings.

### 2.4 Family 4

**Fig. 4:**
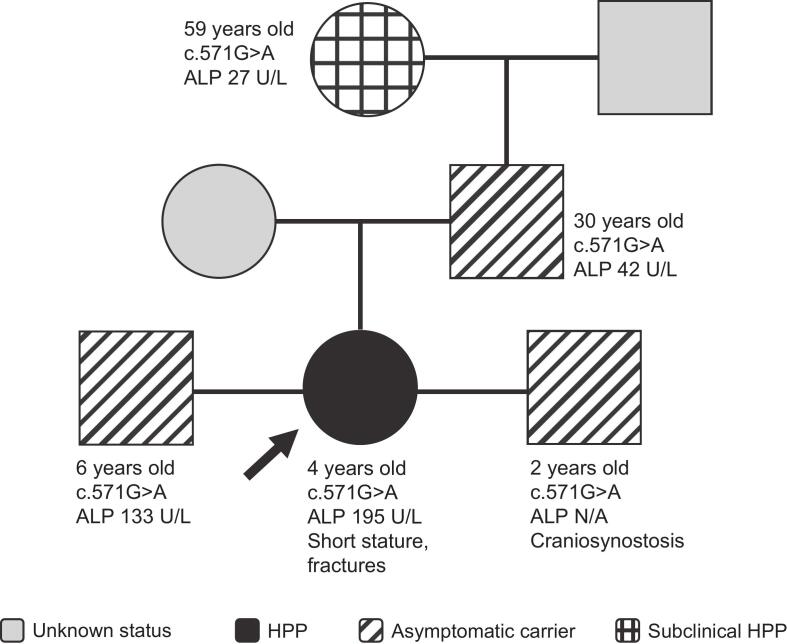
Family 4 pedigree. Patient age at time of assessment, *ALPL* variant, ALP activity (if known), and clinical manifestations are shown on the pedigree for each patient. Arrow indicates proband.

The proband is a 4-year-old girl who was referred for short stature (Fig. 4). Her height at time of assessment was 91 cm (<1st percentile), and her weight was 11.3 kg (<1st percentile). She had a history of three peripheral fractures, including one with minimal trauma. She denied musculoskeletal pain, and radiographs did not show rickets in the metaphyseal region. Her ALP activity was 195 U/L (reference range: 130–430 U/L) and vitamin B_6_ concentration was 185 nmol/L (reference range: 35–110 nmol/L). Genetic testing revealed that she had a heterozygous c.571G>A variant in *ALPL*, and she was diagnosed with HPP based on clinical findings, elevated vitamin B_6_ levels, and genetic test results.

After diagnosis of the proband, genetic testing was performed for her two brothers (aged 6 and 2 years), her father, and her maternal grandmother. Each of these family members had the same heterozygous c.571G>A variant in *ALPL*. The proband's 6-year-old brother was 110 cm tall (11th percentile) and weighed 28 kg (12th percentile) at time of assessment and had no symptoms. His ALP activity was 133 U/L. The proband's 2-year-old brother was 89 cm tall (13th percentile) and weighed 13 kg (27th percentile) at time of assessment and had subtle bilateral coronal suture narrowing (craniosynostosis), suggesting early partial fusion. Surgery was not needed. ALP activity and vitamin B_6_ assessment were not performed for the proband's youngest brother. Her father did not have vitamin B_6_ assessment; his ALP activity was 42 U/L (reference range: 40–120 U/L). He had no symptoms consistent with HPP. The proband's grandmother had low ALP activity (27 U/L [reference range: 40–120 U/L]) and an elevated vitamin B_6_ concentration (565 nmol/L [reference range: 35–110 nmol/L]) but was deemed healthy. The proband's grandmother was considered to have subclinical HPP, while the proband's other assessed family members were considered to be asymptomatic carriers.

### 2.5 Family 5

**Fig. 5:**
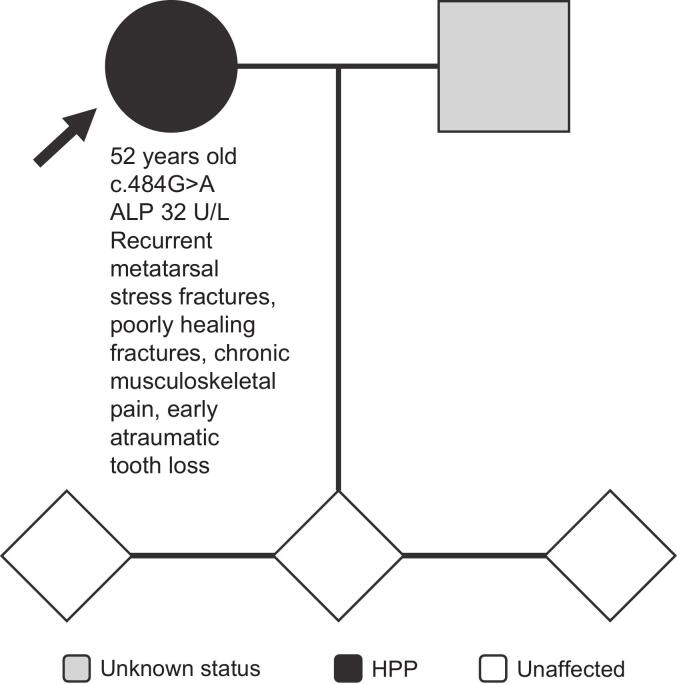
Family 5 pedigree. Patient age at time of assessment, *ALPL* variant, ALP activity, and clinical manifestations are shown on the pedigree for each patient. Arrow indicates proband.

The proband is a 52-year-old woman who had a history of recurrent metatarsal stress fractures, as well as fractures of the ribs and arm. Her fractures were poorly healing. She also reported a history early atraumatic tooth loss, chronic musculoskeletal pain, vertebral (T8) and feet deformity, and osteoporosis (bone mineral density T-score: −2.6) (Fig. 5). She was 165 cm tall and weighed 79 kg at time of assessment, and her ALP activity was 32 U/L. Genetic testing showed that she had a heterozygous c.484G>A variant in *ALPL*, and she was diagnosed with HPP based on clinical, biochemical, and genetic findings. Genetic testing was performed on each of her three children, all of whom were negative for any *ALPL* variant. Each of the three children was asymptomatic and did not have HPP.

### 2.6 Family 6

**Fig. 6:**
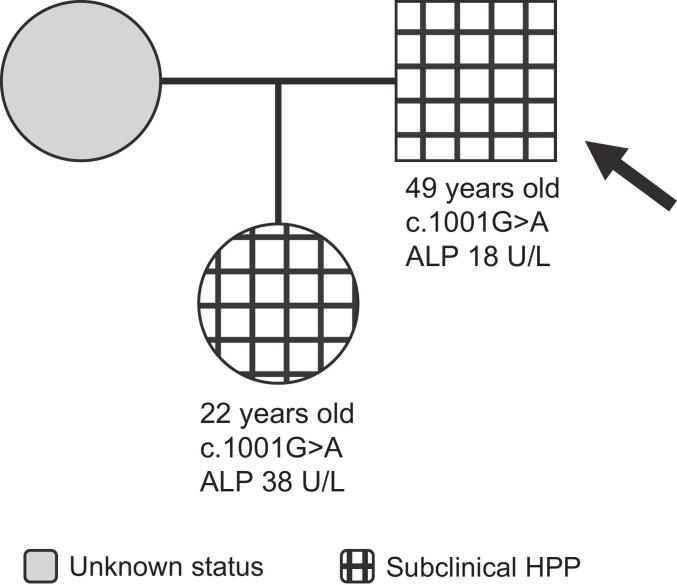
Family 6 pedigree. Patient age at time of assessment, *ALPL* variant, and ALP activity are shown on the pedigree for each patient. Arrow indicates proband.

The proband is a 49-year-old man who was 175 cm tall and weighed 96 kg at time of assessment (Fig. 6). He presented with normal dentition and motor function and denied any nonspecific pain. The proband had been referred for genetic assessment to rule out inherited aortopathy. He had incidental detection of low ALP (18 U/L [reference range, 30–130 U/L]) and a heterozygous c.1001G>A variant in *ALPL*. The proband's 22-year-old daughter (height, 161 cm; weight, 59 kg) had low ALP activity (38 U/L [reference range, 40–120 U/L]) and carried the same heterozygous variant. She was a competitive athlete at time of assessment and was unaffected. The proband and his daughter were considered to have subclinical HPP.

## 3 Discussion

This observational study exemplifies genetic and clinical heterogeneity among and between families with HPP. One *ALPL* variant was detected in each family assessed, and different *ALPL* variants were detected across all families. Within families, heterogeneous manifestations of HPP were observed in family members with the same genotype. Individuals with HPP inherited an *ALPL* variant from a parent who may or may not have had clinical manifestations.

Previous pedigree analyses in families with HPP have shown a similar degree of heterogeneity within and between families, reinforcing the wide array of phenotypes present in those with the disease (Huggins et al., 2020; Hofmann et al., 2014). In addition, some case reports have documented manifestations associated with the specific *ALPL* variants documented in this report. Consistent with the results presented here, previous case reports showed that patients with heterozygous c.1001G>A *ALPL* variants do not demonstrate symptoms of HPP (Huggins et al., 2020; Hofmann et al., 2014). However, it is noteworthy that symptoms are often present when the c.1001G>A *ALPL* variant is combined with another variant (e.g., c.[571G>A];[1001G>A], c.[571G>A];[571G>A]) (Huggins et al., 2020; Hofmann et al., 2014; Leung et al., 2013). In the current study, members of family 4 carried heterozygous c.571G>A variants, although only one family member was diagnosed with HPP, suggesting incomplete genetic penetrance. The c.571G>A *ALPL* variant is common in both the general population and in those with HPP, despite its low predicted enzyme activity (Del Angel et al., 2020; Kishnani et al., 2024). Although the outcomes associated with these variants were consistent between families in the current study and those in previous reports, other variants reported in this study were less consistent with those reported in previous publications. For example, the results of the current study show that the c.1426G>A *ALPL* variant was associated with fractures and osteoporosis in family 1; however, this variant was previously reported in an asymptomatic individual with subclinical HPP (Huggins et al., 2020). The c.484G>A variant was associated with fractures in the current study in family 5 but was previously associated with delayed walking and muscle weakness (Saraff et al., 2016).

This study has some limitations. Although the cases presented herein provide insight into clinical outcomes in families with 6 unique genotypes, several hundred *ALPL* variants have been reported (Farman et al., 2024). Thus, limited conclusions can be drawn about genotype-phenotype correlations in HPP from this case series alone. There remains a need for large-scale efforts to study these correlations in larger cohorts of patients with HPP. In addition to the limited number of variants assessed, all patients in this analysis were heterozygotes. Any interpretation of these results is limited to those with monoallelic HPP. Compared with heterozygous patients, patients with >1 *ALPL* variant (i.e., biallelic disease) are typically more likely to have early-onset disease (Whyte et al., 2015; Kishnani et al., 2024).

The results presented in this study show that families with HPP may have a wide variety of clinical outcomes, including subclinical HPP in some family members. Further, the results of this paper underscore the importance of family mapping as part of routine clinical practice for patients with HPP. A database of *ALPL* variants among patients with HPP (https://alplmutationdatabase.jku.at/) is publicly available and can serve as a useful resource for clinicians to identify manifestations associated with specific variants (Farman et al., 2024). Clinicians are also encouraged to submit any novel variants to the database. Family members of identified probands should receive careful clinical evaluation over time and may potentially undergo genetic testing for familial *ALPL* variants, although genetic testing is not required for a diagnosis of HPP (Khan et al., 2024). This evaluation may be important for early diagnosis of HPP, helping ensure appropriate and timely care for all family members. Individuals with subclinical HPP may need periodic monitoring to assess for potential disease progression.

## CRediT authorship contribution statement

**Peter Kannu:** Writing – review & editing, Writing – original draft, Validation, Investigation, Data curation. **Aliya A. Khan:** Writing – review & editing, Writing – original draft, Validation, Investigation, Data curation. **Mira Francis:** Writing – review & editing, Writing – original draft, Supervision, Project administration, Conceptualization. **Jonathan D. Adachi:** Writing – review & editing, Writing – original draft, Validation, Investigation, Data curation.

## Declaration of competing interest

PK has received consulting fees from Alexion and Ipsen, and his institution has received grants from the Canadian Institutes of Health Research. AAK has received payment/honoraria from Amgen, Calcilytix, and Alexion and meeting support from Ascendis; she is on a data safety or advisory board for Amgen and Amolyt and is the HPP diagnostic criteria chair. MF is an employee of and may own stock/options in Alexion, AstraZeneca Rare Disease; she is on a data safety or advisory board for Alexion, AstraZeneca Rare Disease. JDA has received grants from Amgen and payment/honoraria and consulting fees from Alexion, Amgen, and Sandoz.

## Data Availability

Data will be made available on request.
