# Corrigendum to “Multigenerational genetic inheritance and clinical characteristics of the rare disease hypophosphatasia in 6 families: A case series” [Bone Rep. 26 (2025) 1–6 (101857)]

**DOI:** 10.1016/j.bonr.2025.101866

**Published:** 2025-08-20

**Authors:** Peter Kannu, Aliya A. Khan, Mira Francis, Jonathan D. Adachi

**Affiliations:** a University of Alberta, 8613 114 Street, Edmonton, AB T6G 1C9, Canada; b McMaster University, 1280 Main St W, Hamilton, ON L8S 4L8, Canada; c Alexion, AstraZeneca Rare Disease, 1004 Middlegate Road, Mississauga, ON L4Y 1M4, Canada

The authors regret an error in Fig. 1 of the manuscript titled ‘Multigenerational genetic inheritance and clinical characteristics of the rare disease hypophosphatasia in 6 families: A case series,’ which shows that in Family 1, the proband's niece carries a c.484G>A genetic variant in *ALPL*. The figure should instead show that the niece carries a c.1426G>A genetic variant in *ALPL*, as is correctly described in the manuscript text.

**Fig. 1:**
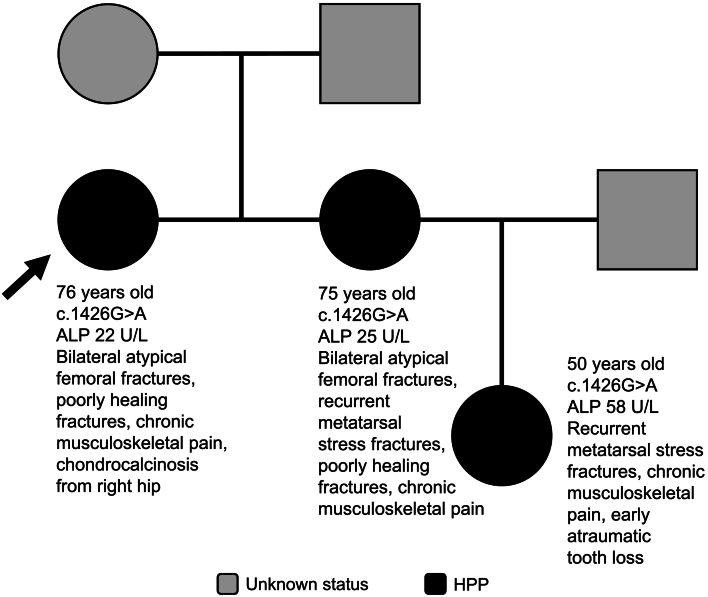

The authors would like to apologise for any inconvenience caused.

